# Alcohol Screening During US Primary Care Visits, 2014–2016

**DOI:** 10.1007/s11606-021-07369-1

**Published:** 2022-01-19

**Authors:** Brittany Chatterton, Alicia Agnoli, Eleanor Bimla Schwarz, Joshua J. Fenton

**Affiliations:** 1 Center for Healthcare Policy and Research, University of California, Davis, 4150 V Street, PSSB 2400, Sacramento, CA 95817 USA; 2 Department of Internal Medicine, University of California, Davis, Sacramento, CA USA; 3 Department of Family and Community Medicine, University of California, Davis, Sacramento, CA USA

**Keywords:** alcohol screening, unhealthy alcohol use, primary care

## Abstract

**Background:**

Unhealthy alcohol use is a significant health issue for the US population. The US Preventive Services Task Force (USPSTF) recommends screening adults 18 years or older for unhealthy alcohol use during primary care visits.

**Objectives:**

To evaluate alcohol screening among ambulatory visits made by US adult primary care patients and identify characteristics predictive of alcohol screening.

**Design:**

A series of cross-sectional analysis of the National Ambulatory Medical Care Survey (NAMCS) data collected from 2014 to 2016 was used to examine US primary care providers’ use of alcohol screening questionnaires and delivery of counseling on alcohol use.

**Participants:**

A total of 19,213 visits made by patients aged 18 years or older to a US primary care physician trained in family medicine or internal medicine.

**Main Measures:**

Administration of a validated alcohol screening questionnaire and counseling/education on alcohol use. Variation in alcohol screening by patient demographic characteristics, reason for office visit, length of office visit, chronic medical conditions, evaluation by assigned primary care physician, new patient to practice, and region.

**Key Results:**

Alcohol screening with a validated questionnaire occurred during 2.6% (95% Cl: 0.9%, 4.3%) of visits. Alcohol counseling, provided either by the physician or by referral, was documented in 0.8% (95% Cl: 0.3%, 1.3%) of visits. Screening was significantly more likely if patients were seen by their assigned primary care physician (adjOR 4.38 (95% Cl: 1.41, 13.61)), a new patient to the practice (adjOR 4.18 (95% Cl: 2.30, 7.79)), or had several chronic medical conditions (adjOR 3.40 (95% Cl: 1.48, 7.78)). Patients’ sex, race/ethnicity, age group, or length of appointment time was not associated with screening for unhealthy alcohol use.

**Conclusions:**

Screening for unhealthy alcohol use using a validated questionnaire is uncommonly performed during US primary care visits. Interventions or incentives may be needed to increase uptake of USPSTF alcohol screening recommendations.

## INTRODUCTION

Unhealthy alcohol use is a major health and economic issue. Excessive alcohol consumption is linked to a greater risk for liver cirrhosis^1^, cardiovascular disease^2^, infections^3^, accidental deaths^4^, dementia^5^, and cancer^6^. In a recent study, 95,158 US deaths were annually attributable to alcohol use^7^. The economic impact of excessive alcohol use is staggering, annually costing the US an estimated $250 billion^8^.

With such broad health and social impacts, identification of unhealthy alcohol use and prevention of alcohol use disorder is key. The US Preventive Services Task Force (USPSTF) first put forth recommendations for screening for unhealthy alcohol use in 1996^9^ (evidence level B) with updates in 2004^10^, 2013^11^, and 2018^12^. These recommendations include screening adults aged 18 years and older with brief standardized screening questionnaire, such as the Alcohol Use Disorders Identification Test (AUDIT), to identify the full spectrum of unhealthy alcohol use, ranging from risky drinking to alcohol use disorder, followed by counseling by the primary care physician if screening indicates a need. The USPSTF updates have reflected new data supporting the use of structured questionnaires and guidance on how counseling is conducted. These recommendations were based on evidence that screening tools are effective in identifying patients at risk for unhealthy alcohol use and counseling completed by a primary care provider is effective in reducing alcohol use^12^. However, the USPSTF has yet to recommend a specific frequency for alcohol screening.

Prior studies of the prevalence of alcohol screening in office visits have relied on patient self-report. The Centers for Disease Control and Prevention (CDC)’s Behavioral Risk Factor Surveillance System (BRFSS) national survey in 2017 found 81% of patients’ self-reported alcohol screening^13^ when asked if during a routine checkup within the prior 2 years a medical professional inquired whether they consumed alcohol. Similarly, a recent analysis of the National Survey for Drug Use and Health (NSDUH) data found 76–87% of participants reported being asked about alcohol use by a medical professional in the past year^14^. There are, however, currently relatively few studies examining clinician reports of screening for unhealthy alcohol use. The aim of this study was therefore to evaluate rates of alcohol screening using recommended structured questionnaires during US primary care physicians’ visits and to identify variables associated with alcohol screening and education.

## METHODS

We conducted a series of cross-sectional analyses of the National Ambulatory Medical Care Survey (NAMCS) data. NAMCS is a nationally representative population-based survey of US ambulatory physicians that utilizes a multistage probability design. Physicians are recruited to join NAMCS by US census regions and a field representative from the census bureau abstracts NAMCS data from the medical chart on at least half the visits conducted during the physician’s pre-selected reporting week. The basic sampling unit for NAMCS is the physician-patient ambulatory visit. Publicly available survey data from 2014, 2015, and 2016 were included in this study. We limited these analyses to visits made by patients aged 18 years or older to physicians practicing family or internal medicine.

We analyzed the proportion of visits in which alcohol screening was completed, as indicated by documentation in the medical record of use of any alcohol screening questionnaire (including AUDIT; Michigan Alcohol Screening Test (MAST); Cut down, Annoyed, Guilty, and Eye-opener(CAGE); Tolerance, Annoyed, Cut down, Eye-opener (T-ACE)), documentation of education/counseling on alcohol by the physician, and/or any referrals placed for alcohol education/counseling. We combined data from 2014 thru 2016 to examine associations of screening for alcohol use with patient characteristics including sex, race/ethnicity, age, and number of chronic medical issues, and visit characteristics including US region, whether patients were new or established with the medical practice, whether the visit was conducted by the patient’s assigned primary care physician, reason for the clinical visit (new problem (present for < 3 months), chronic problem (flare or routine), preventative care, or pre/post-surgery), and length of time spent with the physician during the encounter.

We created multivariable models to examine the odds of alcohol screening and/or counseling occurring during a primary care visit after controlling for patient sex, race/ethnicity, age, reason for visit, number of chronic conditions, being a new patient to the practice, being seen by one’s assigned primary care physician, visit length, US region where the office visit occurred, and year of the visit. The alcohol screening/counseling variable was created by combining the NAMCS variables ETOH and ETOHED.

All data analysis was completed with SAS statistical software, version 9.4 (SAS Institute, Cary, NC, USA), using PROC SurveyFreq to obtain weighted percentages of the frequency of the variables of interest with 95% confidence intervals and the NOMCAR procedure to address missing data (3 variable with < 5% missing data) as recommended by the National Center for Health Statistics (NCHS)^15, 16^. Statistical modeling was completed with multivariable logistic regression utilizing PROC SurveyLogistic to properly account for the complex survey design. Significance level was set at an alpha level of 0.05. This study utilized publicly available data and thus was exempt from institutional review board approval.

## RESULTS

We identified 19,213 visits to participating primary care providers between 2014 and 2016. Most visits lasted less than 30 min. Less than a third of visits were for preventative care and most were conducted by the patient’s assigned primary care physician. Additional patient and visit characteristics are shown in Table 1.

**Table 1:** Demographic and clinical characteristics of patients seen in visits to US primary care physicians (*N* = 19,213)

	Percentage* (*n*)
Sex	
Male	42.9% (8145)
Female	57.0% (11068)
Age (years)	
18–24	5.6% (1112)
25–34	9.7% (1855)
35–44	12.2% (2390)
45–54	17.9% (3310)
55–64	20.9% (3979)
≥ 65	33.5% (6567)
Race/ethnicity**	
White, non-Hispanic	67.0% (14,454)
Black	12.6% (2,039)
Hispanic	14.2% (1,757)
Other and multiracial	6.0% (963)
Number of chronic conditions	
0	22.8% (4,606)
1–2	41.8% (8,047)
≥ 3	34.1% (6,339)
Missing	1.1% (221)
Region of office visit	
Northwest	17.9% (2,741)
Midwest	22.7% (5,552)
South	38.6% (6,826)
West	20.6% (4,094)
Has the patient been seen in the practice before?	
Yes, established patient	90.1% (17,177)
No, new patient	9.8% (2,036)
Patient seen by assigned primary care physician	
Yes	86.9% (15,994)
No	9.3% (2,139)
Missing	3.6% (1,080)
Visit type	
New problem (<3 months onset)	34.6% (6,985)
Chronic problem (routine or flare-up)	39.1% (7,508)
Pre/post-pre/post-surgery	2.4% (377)
Preventative care	21.5% (3,924)
Missing	2.1% (419)
Time spent with the physician	
≤ 10 min	8.4% (1,569)
11–19 min	41.1% (8,073)
20–29 min	26.3% (5,108)
30–41 min	15.7% (3,087)
≥ 40 min	8.2% (1,376)

*Weighted percentages

**Imputed, raw data 19.6% missing

Alcohol screening with a questionnaire (i.e., AUDIT, CAGE, MAST, T-ACE) was documented in 2.6% (95% Cl: 0.9%, 4.3%) of visits. Alcohol counseling, provided either by the physician or by referral to a substance use specialist, was documented in 0.8% (95% Cl: 0.3%, 1.3%) of visits, including some in which there was no alcohol screening with a questionnaire. Patients were significantly more likely to be screened for alcohol if they were seen by their assigned primary care physician, a new patient to the practice, or had chronic medical conditions (Table 2). Patients were significantly less likely to receive alcohol screening if they were being seen for a new problem or a pre/post-surgical appointment compared to those being seen for a preventative care visit. Regional differences were noted with screening less likely to occur in the Midwest and South compared to the West (Table 2). Patients’ sex, race/ethnicity, age group, or length of appointment time was not associated with screening for unhealthy alcohol use.

**Table 2:** Variables associated with addressing alcohol use* in US primary care, 2014–2016 NAMCS (*N* = 17,617 visits)

Patient and visit characteristics	Adjusted** OR	95% CI
Sex		
Male	1.0	
Female	1.09	0.81, 1.47
Race/ethnicity		
White	1.0	
Black	0.83	0.53, 1.26
Hispanic	1.33	0.67, 2.66
Other and multiracial	0.32	0.08, 1.21
Age (years)		
≤ 24	1.0	
25–34	1.24	0.48, 3.16
35–44	1.10	0.44, 2.75
45–54	0.94	0.38, 2.31
55–64	0.89	0.35, 2.26
≥ 65	0.84	0.34, 2.08
Number of chronic conditions		
0	1.0	
1 to 2	1.81	1.00, 3.27
≥ 3	3.40	1.48, 7.78
New patient to practice		
No, established patient	1.0	
Yes, new patient	4.18	2.30, 7.59
Seen by assigned primary care		
No	1.0	
Yes	4.38	1.41, 13.61
Reason for visit		
Preventative care	1.0	
New problem (<3 months onset)	0.43	0.21, 0.88
Chronic problem (routine or flare)	0.71	0.40, 1.26
Pre/post-surgery	0.03	0.006, 0.23
Visit time		
≤ 10 min	1.0	
11–19 min	2.10	0.47, 9.46
20–29 min	1.69	0.34, 8.46
30–39 min	0.80	0.17, 3.77
≥ 40 min	1.14	0.20, 6.42
US region		
West	1.0	
Northeast	1.04	0.40, 2.72
Midwest	0.12	0.04, 0.38
South	0.28	0.09, 0.85
Year		
2014	1.0	
2015	3.5	1.51, 8.14
2016	4.0	1.64, 10.04

*Defined as screening for unhealthy alcohol use with a validated questionnaire, counseling on alcohol, and/or referral for alcohol counseling

**Adjusted for sex, race/ethnicity, age, number of chronic medical conditions, being new to the primary care practice, being seen by one’s assigned primary care physician, reason for office visit, length of visit time with the physician, US region of where the office visit occurred, and year of screening

## DISCUSSION

In this nationally representative sample of US primary care office visits, we found that despite current USPSTF recommendations, screening for alcohol use disorder was documented during less than 3% of ambulatory patient encounters. Regional differences were identified with screening occurring more frequently in the West compared to the Midwest and South. Visits for new problems or pre/post-surgery were significantly less likely to include screening than preventative care visits. Although it would be expected that preventative care visits would have higher screening rates, surgical patients with alcohol use disorders are at particularly higher risk for perioperative complications including sepsis, acute respiratory distress syndrome (ARDS), cardiovascular events, and pneumonia^17, 18^. Thus, particular attention should be given to screening patients for alcohol use during pre-operative evaluations by primary care physicians. Patients seen by their assigned primary care physician were significantly more likely to receive this recommended screening. This may be because primary care physicians feel more responsible for performing preventive screenings for their assigned panel of patients or because they have more established trusting relationships which facilitate discussion of sensitive topics.

The low rates of documented screening contrast with BRFSS and NSDUH data indicating that around 80% of US adults report having been screened for alcohol use by a clinician in the prior 1 to 2 years^13, 14^. This difference is likely due in part to the fact that the current study assesses documented screening with a validated screening questionnaire, whereas both BRFSS and NSDUH assess whether patients recall having been asked by a clinician if they drank alcohol. In addition, the current study also focuses on primary care visits while the BRFSS and NSDUH ask respondents about their experiences with all medical care providers.

The utility of using validated screening questionnaires arises from improved sensitivity and specificity of identifying patients across the full spectrum of unhealthy alcohol use from risky drinking (i.e., binge drinking) to alcohol use disorder^19^. Of the questionnaires available, the USPSTF 2018 update recommends use of AUDIT-consumption(AUDIT-C) (How often did you have a drink containing alcohol in the past year?, How many drinks containing alcohol do you have on a typical day when you were drinking?, How often did you have 6 or more drinks on one occasion?) or the Single Alcohol Screening Question (SASQ) (How many times in the past year have you had 5 (for men) or 4 (for women and all adults older than 65) or more alcohol containing drinks in one day)^12^. If positive by initial 1–3 question screening, then further questionnaires, such as the complete AUDIT, should be asked. Of note, although CAGE is a popular questionnaire among primary care physicians, it identifies alcohol dependence, without effectively screening for risky drinking^12^.

The low rates of structured screening for alcohol use in NAMCS may also stem from the fact that the NAMCS data is collected during a pre-selected reporting week. As such, patients could have been screened during a prior or later visit not captured by NAMCS. As, on average, US patients see a physician less than 3 times a year^20^, our findings indicate that if screening using a validated questionnaire occurred during only 2.6% (95% CI: 0.9%, 4.3%) of each visit, then over the course of 1 year, less than 12.9% of US primary care patients would receive such screening. While annual preventative care visits with a trusted primary care physician may increase the likelihood of screening and reduce the adverse health consequences of excessive alcohol use, it has been estimated that primary care physicians would need to spend more than 7.5 hours with each patient to address all issues recommended by the USPSTF^21^. A recent systematic review found time constraints and competing demands were key barriers to screening and brief intervention for alcohol use in primary care settings^22^, and many primary care physicians may find it difficult to routinely administer structured questionnaires. An additional barrier leading to low screening rates could also stem from physicians feeling there is not sufficient access to treatment for alcohol use disorders; thus, identification of alcohol use disorders could lead to feeling under-resourced to provide sufficient treatment to their patients.

There are limitations to the study. Due to the cross-sectional nature of NAMCS, it is possible that some patient may have been screened during a previous or subsequent primary care visit. As such, rates of screening with a validated alcohol screening questionnaire may be underestimated; however, even if the identified screening rate was one or two magnitudes greater, it would still be far below an optimal screening rate. Additionally, data abstracted from the medical chart would not identify if screening with a validated questionnaire was completed but not documented in medical records. Finally, data regarding if a patient abstains from alcohol is not available within NAMCS; thus, some patient may have reported abstaining from alcohol and thus were not screened using a validated questionnaire. However, given the 86% lifetime prevalence of alcohol consumption among US adults^23^, the majority of patients in primary care settings would qualify for screening.

In conclusion, in this nationally representative sample of US primary care office visits, we found that despite current USPSTF recommendations, structured screening for risky drinking rarely occurs. Considering the importance of screening adults for unhealthy alcohol use, strategies that avoid the direct reliance on the primary care physician deserve consideration to help increase screening rates. Technology-based alcohol screening strategies^24^ including web-based screening and brief intervention have been shown to successfully identify and reduce alcohol consumption^25^. Web-based screening could be feasible for primary care practices that have patient web portals and could be completed prior to upcoming appointments^26^. For those practices without web-based platforms, screening questionnaires could be administered in the waiting room prior to the office visit. These strategies offer a way to screen patients without overtaxing limited primary care visit time.
